# Metastasis of breast cancer to the right kidney with a tumor thrombus in the inferior vena cava: a case report

**DOI:** 10.1186/s40792-022-01364-2

**Published:** 2022-01-17

**Authors:** Ayako Nagata, Yoshiaki Shinden, Yuki Nomoto, Hazuki Saho, Akihiro Nakajo, Koji Minami, Yuichi Kumagae, Mari Kirishima, Tetsuhiro Owaki, Takao Ohtsuka

**Affiliations:** 1 Department of Digestive Surgery, Breast and Thyroid Surgery; Graduate School of Medicine and Dental Sciences, Kagoshima University, 8-35-1 Sakuragaoka, Kagoshima-City, Kagoshima 890-8520 Japan; 2 Department of Radiology, Graduate School of Medical and Dental Sciences, Kagoshima University, 8-35-1 Sakuragaoka, Kagoshima-City, Kagoshima 890-8520 Japan; 3 Department of Pathology, Graduate School of Medicine and Dental Sciences, Kagoshima University, 8-35-1 Sakuragaoka, Kagoshima-City, Kagoshima 890-8520 Japan; 4 Department of Community-Based Medicine, Education Center for Doctors in Remote Islands and Rural Areas, Kagoshima University, 8-35-1 Sakuragaoka, Kagoshima City, Kagoshima 890-8520 Japan

**Keywords:** Breast cancer, Kidney metastasis, Inferior vena cava thrombus

## Abstract

**Background:**

It is quite rare for breast cancer to metastasize to the kidney with a tumor thrombus in the inferior vena cava.

**Case presentation:**

A Japanese woman in her forties was diagnosed with cancer of the left breast and underwent left mastectomy with sentinel lymph node biopsy. The final pathological diagnosis was pT1aN0M0, stage IA (ER positive, PgR positive, HER2 negative). Thirteen years later, she presented for care with the complaint of abdominal pain. By imaging findings, right renal carcinoma with a tumor thrombus in the inferior vena cava and lung metastases was suspected. However, her tumors were refractory to molecular targeted therapy. In addition, CT-guided needle biopsy of the kidney and lung lesions was done and it was revealed that lesions of the left lung and the right kidney was breast cancer metastases (ER positive, PgR positive, HER2 negative). The patient started combination therapy consisting of abemaciclib, tamoxifen and leuprorelin. Six months later, she died from progression of her metastatic disease.

**Conclusions:**

It is sometimes difficult to differentiate between primary renal cancer and kidney metastases from breast cancer on imaging. Renal biopsy is recommended before commencing treatment.

## Background

Breast cancer remains the most lethal malignancy in women worldwide [1]. Mortality from breast cancer is associated with metastatic spread. The sites of metastasis vary, and the most common sites are lymph nodes, bone, lung, brain, and liver [2]. However, the number of breast cancer survivors increases with advances in diagnosis and treatment, the development of secondary malignancies and unusual metastatic patterns is being frequently recognized [3]. Microscopic metastases of primary breast cancer to the kidney are commonly found at autopsy with an incidence of 12.6% [4]. Yet clinically apparent renal metastasis from breast cancer is rare and there are several case reports describing the radiologic findings and symptoms of kidney metastasis from breast cancer [2, 3, 5, 6]. In addition, to the best of our knowledge, there are no prior reports of kidney metastasis from breast cancer presenting with a thrombus in the inferior vena cava. It is sometimes difficult to tell the differences on imaging between primary cancer of the renal pelvis and a metastatic lesion. Herein, we showed a breast cancer patient with metastases of kidney and tumor thrombus in inferior vena cava and the necessity of biopsy before treatment for presumed renal cancer.

## Case presentation

**Fig. 1:**
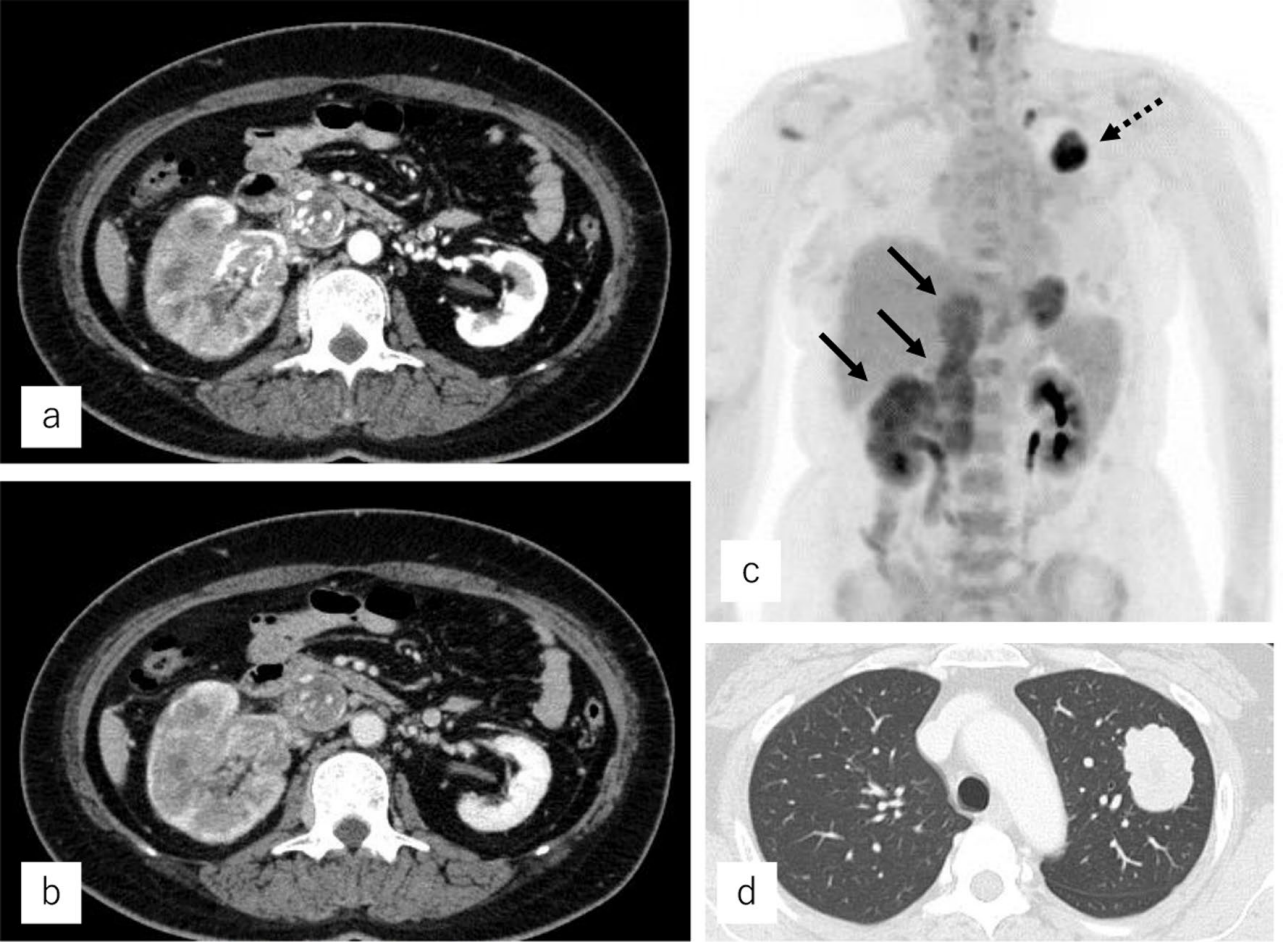
Contrast-enhanced CT. A right renal tumor is occupying the entire right kidney and protruding outside the cortex. There is a tumor thrombus visible in the inferior vena cava on early phase (**a**) and late-phase (**b**) imaging. Positron emission tomography–CT shows kidney metastasis with a tumor thrombus in the inferior vena cava (arrows) and lung metastases (dashed arrow) (**c**). Plain CT shows a tumor in the left lung (**d**)

**Fig. 2:**
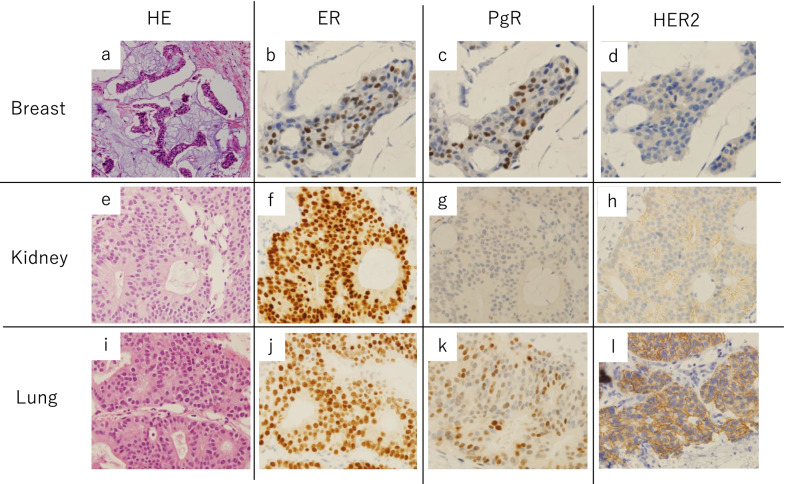
Pathological findings. Almost of all breast cancer lesions had intraductal cribriform structures. There was small invasive lesions with mucus representing stromal infiltration (**a**, HE ×200). ER positive (**b**), PgR positive (**c**) and HER2 negative (1+) (**d**). Renal tumor had proliferative tumor cells with ductal and cribriform structures, and the tumor cells are similar to those of breast cancer (**e**, HE ×400). ER positive (**f**), PgR positive (**g**) and HER2 negative (2+, FISH negative) (**h**) for renal tumor. Cells in lung tumor were also similar to those of breast cancer (**i**, HE ×400). ER positive (**j**), PgR positive (**k**) and HER2 negative (2+, FISH negative) (**l**) for lung tumor. Both TTF-1 and Napsin-A were negative (not shown)

A Japanese woman in her forties visited a nearby clinic because of lower abdominal pain. Thirteen years prior, she had undergone a left mastectomy with sentinel lymph node biopsy for breast cancer; the pathological diagnosis was invasive ductal carcinoma, pT1aN0M0, stage IA. Her tumor was positive for ER and PgR, and negative for HER2. She received tamoxifen plus goserelin for 2 years as adjuvant therapy. Contrast-enhanced CT showed masses in her left lung and a mass in her right kidney, with an apparent tumor thrombus in the inferior vena cava. The renal tumor displayed hypovascular features in both the early and late phases, exhibited diffuse and invasive growth, and occupied the entire right kidney (Fig. 1a, b). She was diagnosed with primary kidney cancer. Based on the imaging findings, we suspected papillary renal cell carcinoma or chromophobe renal cell carcinoma more strongly than clear cell renal cell carcinoma because of the hypovascular and homogeneous features present in our patient’s lesion. The tumor thrombus in the vena cava reached to the height of the diaphragm, so it was impossible to insert a venous filter. Although surgical resection was considered, the lesion was too large. Instead, molecular targeted therapy consisting of nivolumab and ipilimumab for renal cell carcinoma was started. Unfortunately, the tumors did not shrink after 3 months of therapy. The patient underwent a CT-guided needle biopsy of the kidney and lung lesions; pathological examination revealed that both organs contained lesions resembling primary breast cancer that were ER positive, PgR positive and HER2 negative (Fig. 2). Other immunohistological examinations such as TTF-1 showed that her tumors were breast cancer metastasis and she was referred to our department. Considering that a pulmonary thrombus could be lethal if the patient’s tumor collapsed as a result of systemic chemotherapy, we selected endocrine therapy consisting of tamoxifen, leuprorelin, and abemaciclib. After a few months of treatment, her hepatic and renal dysfunction worsened, and she was unable to continue systemic therapy. She died 6 months after starting systemic treatment.

## Discussion

Metastases to the kidney are often diffusely invasive and form small, multiple, bilateral, circular- or wedge-shaped lesions that rarely protrude outside the kidney capsule [7]. The most common form of kidney cancer, clear cell carcinoma, has smooth edges and a uniform internal structure. However, when the tumor grows, it sometimes forms irregular edges, developing an uneven internal structure and cystic features [8]. Contrast-enhanced CT shows a hypervascular lesion with a high degree of contrast effect in the early phase, and lower absorption than the surrounding normal renal parenchyma in the late phase. In contrast, papillary renal cell carcinoma and chromophobe cell carcinoma, which are atypical renal cancers, have hypovascular characteristics on contrast-enhanced CT [9]. Our patient had a kidney lesion that formed a diffusely invasive, large, single mass that protruded outside the cortex. While invasion into the inferior vena cava from a renal metastasis of breast cancer has not been previously reported, tumor thrombus in the renal vein and inferior vena cava reportedly occurs up to 10% of patients with renal cell carcinoma [10, 11]. Therefore, renal lesion was initially diagnosed as renal cell carcinoma in this case. Recently, the use of renal tumor biopsy has been rapidly increasing due to improvements in safety and accuracy of diagnosis. However, traditionally, renal tumor biopsy is only performed for selected patients in whom pathological information is needed for treatment decisions [12]. Renal biopsy is recommended when kidney masses are suspicious for metastatic disease in the presence of a known extrarenal malignancy [12]. In our patient, her early breast cancer was 13 years prior, so she was considered to have no known current extrarenal malignancy. It is our opinion that kidney biopsy is required for a definitive diagnosis in patients with a kidney lesion and a tumor thrombus in the inferior vena cava, even though this is a very rare presentation in a patient who had early stage breast cancer 13 years prior.

## Conclusions

It is sometimes difficult to distinguish between primary renal cancer and metastatic breast cancer to the kidney. Multidisciplinary collaboration among clinicians and renal biopsy is recommended for correct diagnosis and appropriate treatments.

## Data Availability

The data are not available for public access because of patient privacy concerns.

## Abbreviations

ER: Estrogen receptor
PgR: Progesterone receptor
HER2: Human epidermal growth factor receptor 2
CT: Computed tomography
HE: Hematoxylin and eosin staining
